# Expansion of influenza A(H1N1)pdm09 NA:S247N viruses with reduced susceptibility to oseltamivir, Catalonia, Spain, and in Europe, July to October 2025

**DOI:** 10.2807/1560-7917.ES.2025.30.48.2500873

**Published:** 2025-12-04

**Authors:** Narcís Saubi, Cristina Andrés, Ignasi Prats-Méndez, Alejandra González-Sánchez, Alysa Davtyan, Rodrigo Vásquez-Mercado, Ariadna Rando, Patricia Nadal, Juliana Esperalba, Maria Arnedo, Marina Vicente, Eva Balada, Jacobo Mendioroz, María Carmen Martín, Karen García-Camuñas, Raquel Vaz, Adrià Najarro, Susana Bernalte, Nieves Larrosa, Andrés Antón, on behalf of the SIVIC Infectious diseases sentinel network

**Affiliations:** 1 Respiratory Viruses Unit, Microbiology Department, Vall d′Hebron Institut de Recerca (VHIR), Vall d′Hebron Hospital Universitari, Vall d′Hebron Barcelona Hospital Campus, Universitat Autònoma de Barcelona, Barcelona, Spain; 2 RELECOV working group, Instituto de Salud Carlos III, Madrid, Spain; 3 Microbiology Department, Vall d'Hebron Institut de Recerca (VHIR), Vall d'Hebron Hospital Universitari, Vall d'Hebron Barcelona Hospital Campus, Universitat Autònoma de Barcelona, Barcelona, Spain; 4 CIBERINFEC, ISCIII-CIBER de Enfermedades Infecciosas, Instituto de Salud Carlos III, Madrid, Spain; 5 General Sub-Directorate for Surveillance and Public Health Emergency Response, Public Health Agency of Catalonia, Barcelona, Spain; 6 https://sivic.salut.gencat.cat

**Keywords:** influenza, S247N, neuraminidase, oseltamivir, A(H1N1)pdm09, Catalonia, Spain

## Abstract

Between July and October 2025, among the total 117 influenza A(H1N1)pdm09 strains characterised in Catalonia, 20% to 100% per week were carrying the NA:S247N substitution. The mutation, conferring reduced susceptibility to oseltamivir, was phenotypically confirmed (IC_50_ between 0.82 and 1.63 nM, compared to median IC_50_ of 0.3 nM for susceptible strains). An increased proportion of S247N variants was also observed in sequence data (10,944 sequences) from other parts of Spain and five of 35 submitting countries across Europe.

During the sentinel surveillance of respiratory viruses in Catalonia, Spain, an increase in the percentage of influenza A(H1N1)pdm09 sequences harbouring the NA:S247N substitution was detected from summer 2025 when compared with previous years [1-5]. This substitution is related to lower oseltamivir susceptibility, and not resistance [6,7]. Here, we present the results obtained from surveillance in Catalonia, together with phenotypic test results of susceptibility to oseltamivir. We compare our results with the presence of the NA:S47N substitution in other European countries, using sequences downloaded from GISAID.

## Presence of NA:S247N in Catalonia

Through the respiratory virus sentinel surveillance network in Catalonia (SIVIC; https://sivic.salut.gencat.cat), influenza viruses — submitted from 37 sentinel primary care centres and 7 hospitals (Hospital Universitari de Bellvitge; Hospital Universitari Germans Trias i Pujol; Hospital Universitari Doctor Trueta; Hospital Universitari Joan XXIII,; Hospital Universitari Verge de la Cinta; Hospital Arnau de Vilanova; Hospital Universitari Vall d’Hebron) distributed across Catalonia — are genetically characterised by whole genome sequencing using the Illumina platform. An in-house bioinformatic application, mitMutFinder [8], detects the amino acid substitutions responsible for resistance to antivirals (according to the list provided by the World Health Organization) [9].

**Figure 1:**
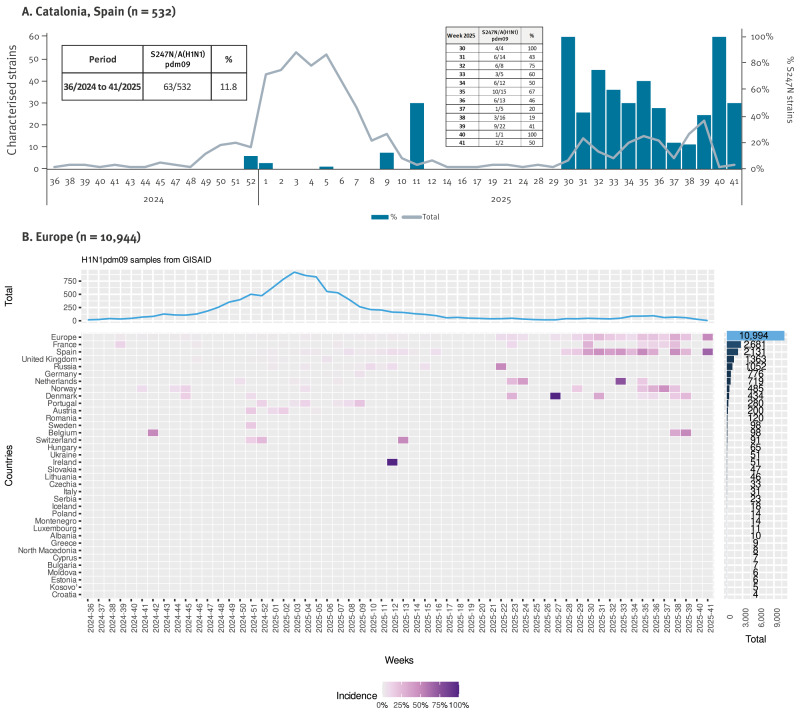
Characterised influenza A(H1N1)pdm09 strains and per cent harbouring NA:S247N substitution, Catalonia, Spain (n = 532) and 35 European countries (n = 10,944), week 36/2024–week 41/2025

^§^ This designation is without prejudice to positions on status, and is in line with UNSCR 1244 and the IСJ Opinion on the Kosovo Declaration of Independence.

An analysis from week 36/2024 to week 41/2025 showed that the prevalence of influenza A(H1N1)pdm09 strains (genetic clades 6B.1A.5a.2a.1 and 6B.1A.5a.2a) carrying the NA:S247N amino acid substitution had increased notably in the last 12 weeks of this period. During the whole period of study, the S247N mutation was detected in 11.8% (63/532) of A(H1N1)pdm09 strains in Catalonia. However, most of the S247N sequences were detected between weeks 30/2025 and 41/2025 (56 S247N/117 A(H1N1)pdm09), with proportions ranging from 20 to 100% (Figure 1A). No I223V substitution, a synergistic substitution that decreases oseltamivir susceptibility when combined to S274N [3], was detected in the sequences from Catalonia.

A. Characterised influenza A(H1N1)pdm09 strains from Catalonia, Spain. The grey line shows the number of weekly influenza A(H1N1)pdm09 characterised strains, and the blue bars show the weekly per cent of strains harbouring the NA:S247N substitution. Embedded tables depict the total number of A(H1N1)pdm09 strains and the number of NA:S247N strains, during the full period (left table; 63/532) or weekly – from week 30/2025 to week 41/2025 (right table; 56/117).

B. A(H1N1)pdm09 strains from 35 European countries downloaded from GISAID, and per cent of sequences harbouring NA:S247N substitution. Upper panel: weekly number of A(H1N1)pdm09 strains (according to sample extraction date). Lower panel: European total and single country percentage of A(H1N1)pdm09 sequences with S247N substitution. Right panel: total number of A(H1N1)pdm09 sequences in GISAID corresponding to each country.

## Distribution of NA:S247N in Europe

To compare our regional findings, influenza sequences from the GISAID database (https://gisaid.org; [10]) from the same study period submitted by European countries were downloaded to assess whether the observed increase in the frequency of S247N A(H1N1)pdm09 strains was limited to Catalonia, or had spread more broadly across Spain and Europe. Analysis of the presence of the S247N mutation in 10,994 sequences of European origin yielded a pattern very similar to ours. Overall, we identified ca 2.2% (241/10,994) of influenza A(H1N1)pdm09 strains with the S247N substitution. However, there was an increase from week 26/2025 onwards in the percentage of influenza A(H1N1)pdm09 sequences containing the S247N substitution, with a maximum of 50% at week 41 (Figure 1B). 

When European results were stratified by country of origin, three different profiles were observed: (i) countries reporting ~100 A(H1N1)pdm09 sequences with an increase of S247N mutation from week 26/2025 onwards, showing a percentage profile similar to that of Catalonia: Spain (including sequences from Catalonia), France, the Netherlands, Norway, Denmark and Belgium; (ii) countries with ~100 of reported A(H1N1)pdm09 sequences, but without an increased S247N proportion: United Kingdom, Russia, Germany, Portugal, Austria, Romania and Sweden; (iii) countries with few A(H1N1)pdm09 sequences reported, i.e. less than 100 A(H1N1)pdm09 sequences.

## Phylogenetic analysis of the samples

**Figure 2:**
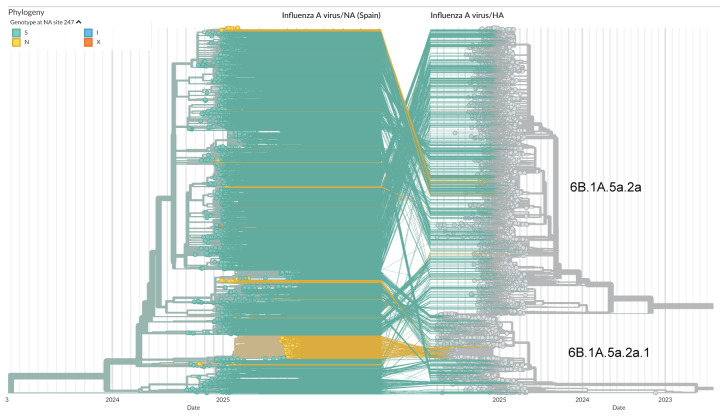
Tanglegram showing influenza A(H1N1)pdm09 phylogenies for neuraminidase and hemagglutinin genes from available sequences in GISAID EpiFlu, September 2024–October 2025 (n = 2,132 NA sequences)

HA: hemagglutinin; NA: neuraminidase.

Neuraminidase (NA) and hemagglutinin (HA) sequences have been presented as a tanglegram to detect any relationship between NA and HA segments (Figure 2). Most S247N sequences cluster together within a well-defined group in the NA phylogeny. However, additional S247N variants were also detected scattered across the NA tree. When mapped to their corresponding HA sequences, these viruses belonged to different genetic clades, predominantly 6B.1A.5a.2a.1 for the main cluster (July–October 2025 sequences) and 6B.1A.5a.2a for the isolates in first months of 2025.

NA sequences (left; 2,132 NA Spanish sequences) and HA sequences (right; 11,100 HA European sequences) available on GISAID EpiFlu (https://www.gisaid.org) sampled between September 2024–October 2025. The NA phylogenetic tree shows all the Spanish influenza A(H1N1)pdm09 sequences in the study period. Dots in green represent NA sequences with the S247 (or wild type), and dots in yellow represent sequences harbouring the S247N substitution. The NA sequence and corresponding HA sequence of each virus are connected by lines. The HA tree represents all the European influenza A(H1N1)pdm09 sequences. NA mutant strains in Spain group in two ways: (i) represented as a distinct S247N cluster corresponding to July–August 2025 sequences, where higher proportions of mutant strains were detected (and clustering also at the HA tree, corresponding to clade 6B.1A.5a.2a.1); and (ii) scattered sequences, prior to July 2025, and corresponding to HA clade 6B.1A.5a.2a. The NA-HA tanglegram was constructed using Nextclade (https://clades.nextstrain.org) and visualised by Auspice (https://auspice.us).

## Phenotypic assay

**Figure 3:**
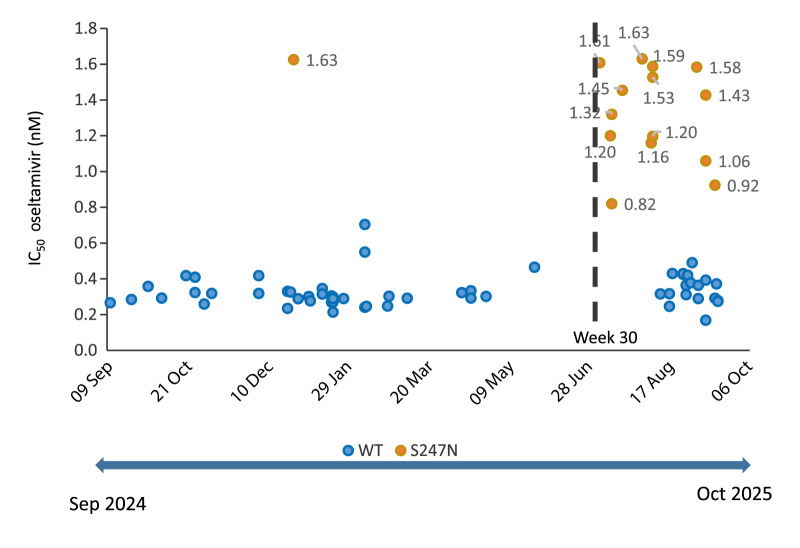
IC_50_ values for oseltamivir in viral isolates with wild-type or S247N substitution strains, Catalonia, Spain, September 2024–October 2025 (n = 74)

In order to confirm that the S247N strains have reduced oseltamivir susceptibility, the MUNANA-based neuraminidase inhibition assay (in-house adapted) [11] was conducted on 74 MDCK-SIAT1 isolates, of which 34 were obtained from week 30 onwards (15 isolates carrying the S247N substitution (confirmed by WGS) and 19 isolates harbouring the S247 residue, the wild-type (WT) sequence with a susceptible phenotype). As previously characterised [6,7], the S247N isolates have higher 50% inhibitory concentration (IC_50_) values for oseltamivir (between 0.82 and 1.63 nM), which is between fourfold and sixfold higher than the WT strains (median 0.3 nM)(Figure 3).

IC_50_: 50% inhibitory concentration.

Viral isolates (n = 74) (MDCK-SIAT1) were tested by the MUNANA-based neuraminidase inhibition assay [11]. The IC_50_ is shown for 16 isolates carrying the S247N substitution (orange) and 58 wild-type isolates (blue) harbouring the S247 residue. From September 2024 to June 2025, there is only one S247N and 39 WT strains. In the period July 2025–October 2025 (from week 30 onwards), 15 S247N and 19 WT were detected. On 31 July, two samples had the same IC_50_ value (1.32) and overlap.

## Discussion

The characteristics of influenza NA:S247 substitutions, and the effects on oseltamivir susceptibility have been described since 2008 [6]. However, the proportion of this substitution in the circulating A(H1N1)pdm09 viruses has never been as high as levels observed in the July–October 2025 period. 

Following the 2009 H1N1 influenza pandemic, the S247N substitution was also described in A(H1N1)pdm09, with similar phenotypic results, i.e. an increase in the oseltamivir IC_50_. In 2011, the S247N substitution was detected in Singapore and Australia, exhibiting a reduction in oseltamivir sensitivity, and an increased proportion of S247N A(H1N1)pdm09 strains, reaching up to 10% of isolates. However, this elevation of mutant strains did not persist, and since then, S247N mutant strains have been reported rarely, either as a single mutation or together with substitutions at the NA:I223 position [7]. 

Toledo-Rueda et al. described only one strain in Mexico during the 2000–17 period [12]. Andrés C et al. reported one case in Spain during 2012–16, an immunocompromised patient undergoing oseltamivir treatment [13]. Similarly, a low number of mutated strains have been described in Bangladesh (2009–12) [14], Brazil (2017–19) [15], Germany (2019–22) [16] and Iran (2010–19) [17]. In Germany in 2023–24, Duwe et al. reported a slight increase, where 1.1% (6/552) of the analysed sequences carried the S247N mutation [3]. In Hong Kong in 2023, Leung et al. reported that 4.35% of influenza A(H1N1)pdm09 sequences were carrying at least one NA substitution related to low antiviral susceptibility and, when compared to GISAID sequences uploaded between September and October 2023, 1.77–2.5% sequences contained S247N [18]. Patel et al. reported 2% sequences with the S247N mutation in the period May 2023–February 2024 in a multicountry study [4]. In the 2023–24 period, our colleagues from the national influenza centre in Valladolid, using GISAID data, reported 2.28% of influenza A(H1N1)pdm09 strains containing S247N mutation [5]. Notably, the World Health Organization, only reported sporadic strains harbouring the S247N mutation in the most recent documents recommending the vaccine composition for the northern and southern hemispheres, respectively [1,19]. Importantly, it has been demonstrated that the S247N strains do not show reduced transmissibility in comparison with strains not carrying this mutation [20].

In our series, the proportion of strains carrying the S247N mutation was much higher, i.e. 20–100% on a weekly basis, with an annual average of 11.8%. Similar results were observed in other Spanish regions and several European countries based on GISAID sequence data. It is important to note that no I223V substitution was detected in the sequences from Catalonia, which, combined with S247N, would further decrease drug susceptibility [21].

## Conclusion

In Europe, the 2025/26 influenza season has started 3–4 weeks early, dominated by A(H3N2) viruses. However, the fact that the proportion of A(H1N1) NA:S247N sequences observed from July to October 2025 was very high in some countries even approaching 100% in some weeks, together with evidence that this mutation does not reduce virus infectivity, warrants strengthening surveillance on the evolution of these A(H1N1) strains.

## Data Availability

Sequence data generated in this study from clinical samples from Catalonia have been shared via GISAID, the global data science initiative, and can be retrieved as data set EPI_SET_251203te (https://doi.org/10.55876/gis8.251203te). Data downloaded from GISAID to generate Figure 1B and Figure 2 can be retrieved as data sets EPI_SET_251203rh (https://doi.org/10.55876/gis8.251203rh) and EPI_SET_251203bs (https://doi.org/10.55876/gis8.251203bs).

## § Note

This designation is without prejudice to positions on status, and is in line with UNSCR 1244 and the IСJ Opinion on the Kosovo Declaration of Independence. 
